# Domain-specific and sex-dependent behavioral alterations in the BTBR mouse model of autism

**DOI:** 10.3389/fnbeh.2026.1932380

**Published:** 2026-09-03

**Authors:** Jacob Jackson, Conlan Houston, Miles Cornils, Ethan Plummer, Chunxiu Yu

**Affiliations:** Department of Biomedical Engineering, Michigan Technological University, Houghton, MI, United States

**Keywords:** anxiety-like behavior, autism spectrum disorder, motor learning, operant learning, social interaction

## Abstract

Autism spectrum disorder (ASD) is a neurodevelopmental condition characterized by social communication deficits and restricted, repetitive behaviors, often accompanied by motor, cognitive, and anxiety-related alterations. Although ASD is diagnosed more frequently in males than females, females remain underrepresented in preclinical studies, limiting understanding of sex-dependent phenotypes. The BTBR T^+^ Itpr3^tf^/J (BTBR) mouse strain is a widely used animal model of ASD, yet its sex-specific behavioral profiles across functional domains remain incompletely characterized. Here, we conducted a systematic, multi-domain behavioral assessment in male and female BTBR mice and wild-type (WT) controls, examining action control, motor learning, social behavior, and anxiety-related exploration. Despite normal acquisition of instrumental responding, BTBR mice exhibited altered action control characterized by reduced habitual responding and persistent sensitivity to action-outcome contingencies following extended habit training, with effects being more pronounced in females. In the accelerating rotarod task, BTBR mice displayed impaired motor learning that was more prominent in males, whereas performance under constant-speed conditions indicated largely preserved baseline motor coordination. Social impairments were dimension-specific: BTBR males showed delayed initiation of social investigation, whereas BTBR females exhibited reduced social engagement. In the elevated plus maze (EPM), overall genotype effects were modest; However, sex-dependent differences emerged within BTBR strain, suggesting altered exploratory behavior and risk-assessment strategies rather than generalized anxiety-like behavior. Together, these findings identify domain- and sex-specific alterations in action selection, motor learning, social behavior and anxiety-related exploration in BTBR mice. These results highlight the importance of sex-stratified analyses in preclinical ASD research and provide a behavioral framework for investigating the mechanisms underlying ASD-relevant phenotypes.

## Introduction

Autism spectrum disorder (ASD) is a neurodevelopmental condition characterized by persistent deficits in social communication and restricted, repetitive patterns of behavior, often accompanied by alterations in motor function, anxiety, and cognitive flexibility (Jiujias et al., 2017; Sellick et al., 2021; Hirota and King, 2023). Importantly, ASD exhibits a pronounced sex bias in prevalence and symptom presentation, with males diagnosed more frequently than females (D’Mello et al., 2022). Accumulating evidence further suggests sex-dependent differences in behavioral phenotypes, symptom severity, and compensatory mechanisms (Werling and Geschwind, 2013). Despite these differences, preclinical studies have historically underrepresented females, limiting understanding of sex as a biological variable in ASD-related behaviors. Therefore, systematically examining sex as a biological variable in preclinical ASD models is critical for improving mechanistic insight and enhancing translational relevance.

The BTBR T^+^ Itpr3^tf^/J (BTBR) mouse strain is a well-established idiopathic model of ASD. This strain displays robust deficits in social interaction, increased repetitive behaviors, alterations in anxiety-like behavior, and impairments in motor and cognitive domains (McFarlane et al., 2008; Meyza et al., 2013; Bove et al., 2024). Neuroanatomical features of BTBR mice, including agenesis of the corpus callosum and altered cortical and striatal connectivity, are thought to underline these behavioral abnormalities (Stephenson et al., 2011; Fenlon et al., 2015; Sforazzini et al., 2016). Although extensive behavioral phenotyping has been performed in BTBR mice, most studies have focused predominantly on males or collapsed data across sexes, potentially overlooking sex-specific behavioral profiles that may be critical for understanding ASD heterogeneity.

Habituation refers to the progressive reduction in behavioral responding to repeated, non-threatening stimuli and represents a fundamental form of learning that supports sensory filtering, behavioral flexibility, and adaptive responding (Ramaswami, 2014; Kepler et al., 2020). Impaired habituation has been implicated in ASD and is thought to contribute to sensory hypersensitivity and repetitive behaviors (Jamal et al., 2021; Shimono et al., 2025). Operant paradigms, such as lever-press tasks, provide a controlled framework to quantify response adaptation and behavioral persistence over time (Yu et al., 2009). However, sex differences in habituation processes within ASD-relevant mouse models remain poorly characterized. In addition to cognitive and adaptive processes, motor coordination, anxiety-like behavior, and social interaction are core domains frequently altered in ASD. Motor differences are highly prevalent and often emerge early in development. Although frequently underrecognized, these impairments can significantly impact social and cognitive functioning (Zampella et al., 2021; da Silva et al., 2025). Anxiety symptoms commonly co-occur in ASD and may differentially affect males and females (Zaboski and Storch, 2018). Social behavior deficits remain a defining characteristic of the disorder, yet emerging evidence suggests that females may exhibit distinct social strategies or compensatory behaviors that mask impairments under certain testing conditions (Klein et al., 2025). Given the multidimensional nature of ASD-related behaviors, comprehensive behavioral assessment across multiple domains is critical. Moreover, evaluating these behaviors in both sexes is essential to accurately model the heterogeneity of ASD phenotypes and to align preclinical research with clinical observations emphasizing sex-dependent differences.

In the present study, we systematically examined sex-specific behavioral alterations in BTBR mice using a series of assays assessing habituation, motor coordination, social interaction and anxiety-like behavior. By directly comparing male and female BTBR mice across these domains, we aimed to determine whether sex modulates the expression of ASD-relevant behavioral phenotypes and to identify behavioral domains particularly sensitive to sex differences in this model.

## Methods

All animal care and experimental procedures were approved by the Institutional Animal Care and Use Committee (IACUC) at Michigan Technological University. The BTBR T^+^ Itpr3^tf^/J (BTBR) mouse strain is an inbred strain that exhibits neuroanatomical and behavioral characteristics relevant to autism spectrum disorder, including agenesis of the corpus callosum and robust deficits in social behaviors (McFarlane et al., 2008). BTBR mice and age-matched C57BL/6J wild-type (WT) mice were obtained from the Jackson Laboratory and maintained through in-house breeding. BTBR mice (*n* = 22, 11 males and 11 females), along with age-matched WT controls (*n* = 20, 10 males and 10 females), were used for all behavioral experiments. WT mice were not littermates of BTBR mice, as BTBR is an inbred strain maintained separately. All animals were housed under identical conditions prior to behavioral testing. The use of C57BL/6J mouse mice as a reference strain for BTBR mouse behavioral phenotyping is widely established in ASD research (McFarlane et al., 2008; Stephenson et al., 2011; McTighe et al., 2013), enabling comparison to a well-characterized baseline across social, cognitive, and motor domains.

All experimental mice were young adults (6–8 weeks of age at the start of testing) to ensure fully developed motor, cognitive, and social behaviors while minimizing developmental variability. Mice were group-housed by sex under standard laboratory conditions (12:12 h light/dark cycle) with ad libitum access to water.

### Instrumental training

For operant training experiments, mice were placed on a food deprivation schedule to reduce their weight to 85% of their baseline weight. After each training session, mice received 1.5–2 g of standard chow, with water available ad libitum in the home cage. Behavioral training was conducted in four operant chambers (15.24 × 13.34 × 12.7 cm, Med Associates) housed within light-resistant and sound-attenuating boxes. Each chamber was equipped with a pellet dispenser delivering 20 mg of food pellets (Bio-Serv). Each chamber contained two retractable levers positioned on either side of a central food magazine and a 3 W, 24 V house light mounted on the wall opposite the levers and magazine. Experimental protocols were controlled and behavioral data were recorded using Med-PC IV software.

#### Lever-press training

At the start of each session, the house light was illuminated, and one lever was inserted into the chamber. At the end of the session, the house light was turned off and the lever retracted. Initial lever-press training consisted of eight consecutive days of continuous reinforcement (CRF), during which mice received one food pellet for each lever press. Sessions ended after 30 rewards or 1 h elapsed, whichever occurred first. Following CRF training, mice were trained under random interval (RI) schedules to promote habitual lever-press behavior (Dickinson et al., 1983; Yu et al., 2009). Mice were first trained for 2 days under an RI-30s schedule, in which reward availability occurred with a 0.1 probability every 30s contingent upon lever pressing. This was followed by 2 days of training under an RI-60s schedule, in which reward availability occurred with a 0.1 probability contingent upon lever pressing.

#### Outcome devaluation

Outcome devaluation was performed using a specific satiety procedure to assess the sensitivity of lever-press behavior to changes in reward value. This approach controls overall satiety and motivational state while selectively altering the value of the earned reward (Yu et al., 2009). Mice were given unrestricted access for 1 h to either the same purified pellets used during lever-press training (devalued condition) or to unlimited home grain food (non-devalued control condition). Consumption of purified pellets reduces the value of the instrumental outcome, whereas the grain food controls for general satiety without devaluing the earned reward. Immediately following the satiety period, mice underwent a 5 min probe test in the operant chamber. During this extinction test, the lever was inserted, but no pellets were delivered. This probe assessed whether lever-press behavior was goal-directed and sensitive to the current value of the outcome (action-outcome association) or whether responding was driven primarily by stimulus-response habits. On the second day of testing, the procedure was repeated with the conditions reversed, such that mice that received home grain food on day 1 received purified pellets on day 2, and vice versa, resulting in a counterbalanced within-subject design.

#### Omission test

Following the devaluation test, mice were retrained under the RI-60s schedule for four additional days. On the following day, the instrumental contingency was reversed using an omission procedure to assess sensitivity to changes in the causal relationship between lever pressing and reward delivery. During the omission session, food pellets were delivered every 20s in the absence of responding. However, each lever press restarted the timer, thereby delaying pellet delivery and establishing a negative contingency between the action and the outcome. Presses per minute were calculated for each 5-min interval. Following the omission test, mice were given full food in their home cage to bring their weight back to their initial weight.

### Rotarod test

Motor coordination and balance were assessed using a rotarod apparatus (Med Associates) as previously described (Wang et al., 2016; Loughlin et al., 2024). On day 1, the rod accelerated from 4 to 40 rpm over a 5 min period. Mice completed four trials, each lasting up to 5 min, with a 30 min inter-trial interval. Trials ended when the mouse fell from the rod or when the maximum duration of 300 s was reached. On day 2, the rod was maintained at a constant speed of 16 rpm, and mice completed four trials under the same conditions as on day 1. Time on the rod was recorded for each trial on both days.

### Social approach test

Each mouse was habituated to an open-field arena (Noldus) for 10 min prior to testing (Wang et al., 2020). After habituation, the mouse was returned to its home cage while the arena was cleaned. An age- and sex-matched stimulus mouse was then placed under an inverted grated pencil holder positioned at the center of the arena. The test mouse was reintroduced for a 10 min social interaction session and tracked using Ethovision XT 17.5 (Noldus). A circular interaction zone was defined around the stimulus mouse to quantify social approach behavior. Measures included time spent interacting with the stimulus, the number of entries into the interaction zone, and latency to first interaction, all of which were recorded and analyzed.

### Elevated plus maze (EPM)

EPM was used to assess anxiety-like behavior in mice (Walf and Frye, 2007). The apparatus (Noldus) consisted of four arms- two open and two enclosed- with a central platform. Each mouse was placed in the center and allowed to explore for 5 min while movements were tracked using Ethovision XT (Noldus). Behavioral measures included latency to closed arms, cumulative time in the closed arms, number of entries into the open arms, and time spent in open arms, all of which were recorded and analyzed.

### Histological analysis

Following completion of behavioral experiments, mice were deeply anesthetized with urethane and perfused transcardially with 0.1 M phosphate-buffered saline (PBS), followed by 4% paraformaldehyde in 0.1 M PBS. Brains were post-fixed overnight in 4% paraformaldehyde at 4 °C, then transferred to 30% sucrose at 4 °C for cryoprotection. Coronal brain sections (40 μm) were cut using a cryostat (Richard-Allan HM 525 NX) and processed for staining. Cytochrome oxidase (CO) staining was performed specifically to verify the characteristic agenesis of the corpus callosum and reduced hippocampal commissure, well-established neuroanatomical features of the BTBR mouse model. The staining was intended to confirm these defining commissural abnormalities rather than provide a comprehensive assessment of neuronal cytoarchitecture or histopathology. Sections were rinsed three times in 0.1 M PBS, then incubated overnight at room temperature in a solution containing 30 mg cytochrome C, 50 mg diaminobenzidine (DAB), 4 g sucrose, and 20 mg catalase per 100 ml of 0.1 M PBS. Following incubation, sections were washed in 0.1 M PBS, mounted onto slides, air-dried, and cover-slipped using Permount mounting medium.

### Data analysis

Statistical analyses were performed to determine significance between groups or conditions. Two-way repeated measures analysis of variance (ANOVA) was used where appropriate. When a significant main effect or interaction was detected (*p* < 0.05), *post hoc* paired comparisons were conducted. To correct for multiple comparisons, pairwise *post hoc*-tests were performed using Turkey’s honestly significant difference (HSD) test with a significance threshold of *p* < 0.05. For single comparisons, the Mann–Whitney U test, paired *t*-test, or unpaired *t*-test was used, as appropriate. Data is presented as mean ± standard error of the mean (SEM), and all individual data points are reported as value ± SEM. Graphs were generated using GraphPad Prism (version 10.4.2).

## Results

### Gross phenotype and histological verification

**FIGURE 1:**
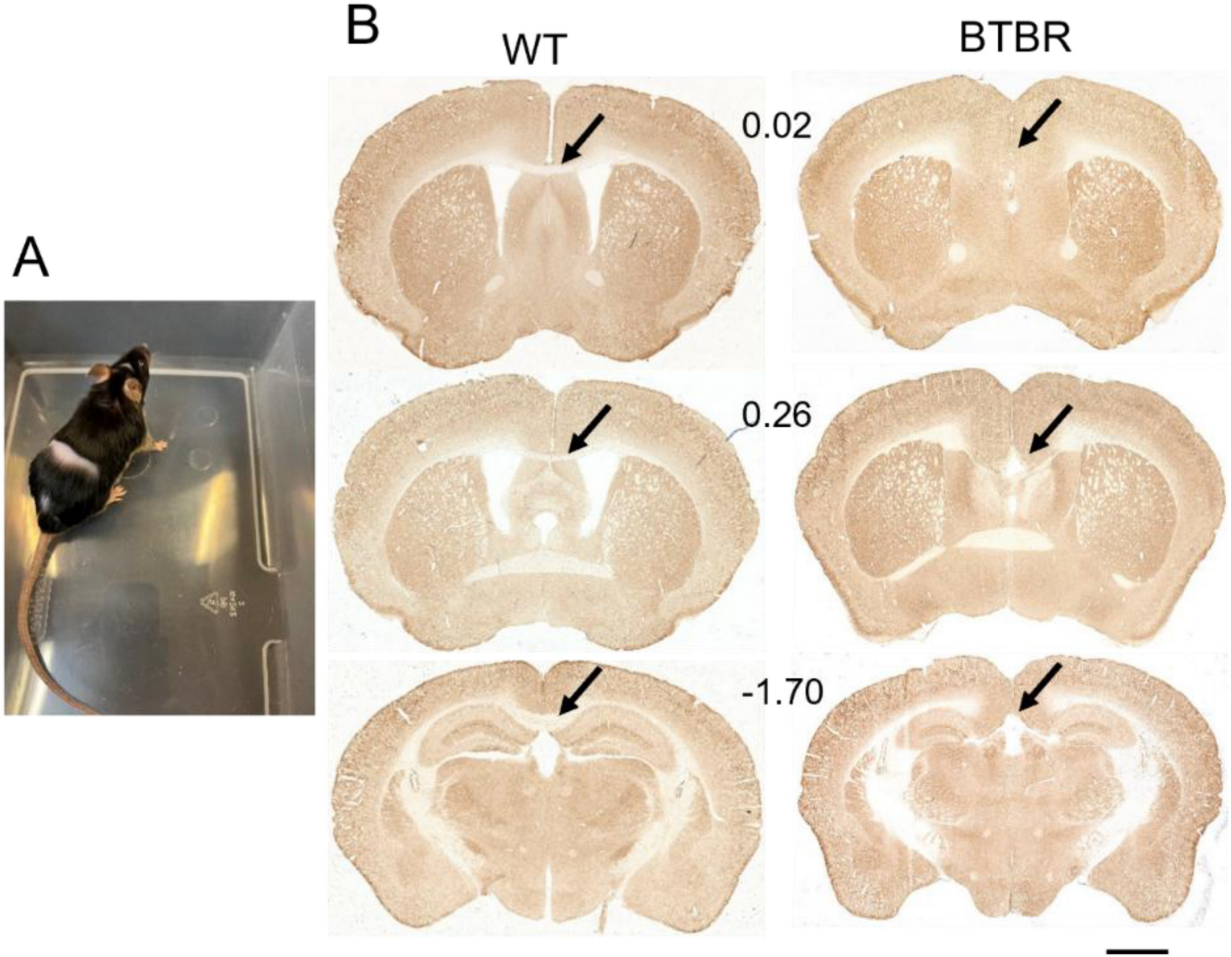
Gross phenotype and histological verification of BTBR mice. **(A)** Representative image of BTBR mice showing overall external appearance. BTBR mice displayed localized hair loss. **(B)** Representative CO-stained coronal brain sections illustrating interhemispheric commissural structures in WT and BTBR mice. BTBR mice exhibit characteristic absence of the corpus callosum and a markedly reduced hippocampal commissure. Sections were collected at matched anterior–posterior levels according to the Paxinos and Franklin mouse brain atlas (Paxions and Watson, 2019). Numbers indicate the approximate distance (mm) from Bregma. Scale bar = 1mm.

BTBR mice exhibited gross physical and neuroanatomical features consistent with previously reported strain-specific characteristics (Stephenson et al., 2011). Compared to WT controls, BTBR animals showed a comparable external appearance overall, with the notable presence of localized hair loss observed in BTBR animals (Figure 1A). No overt differences in body posture or general health status were detected between genotypes during routine handling. Histological examination revealed clear differences in brain structure between BTBR and WT mice. As expected, BTBR mice displayed marked abnormalities in midline commissural structures, including an absence of the corpus callosum and a severely reduced hippocampal commissure. In contrast, WT mice exhibited intact interhemispheric connections with normal corpus callosum morphology (Figure 1B). These structural alterations were consistently observed across BTBR samples and aligned with established neuroanatomical features of this strain. Together, the gross phenotypic observations and histological findings confirm the characteristic neurodevelopmental abnormalities of BTBR mice while indicating no unexpected morphological deviations that would confound subsequent behavioral analyses.

### Initial acquisition (CRF)

**FIGURE 2:**
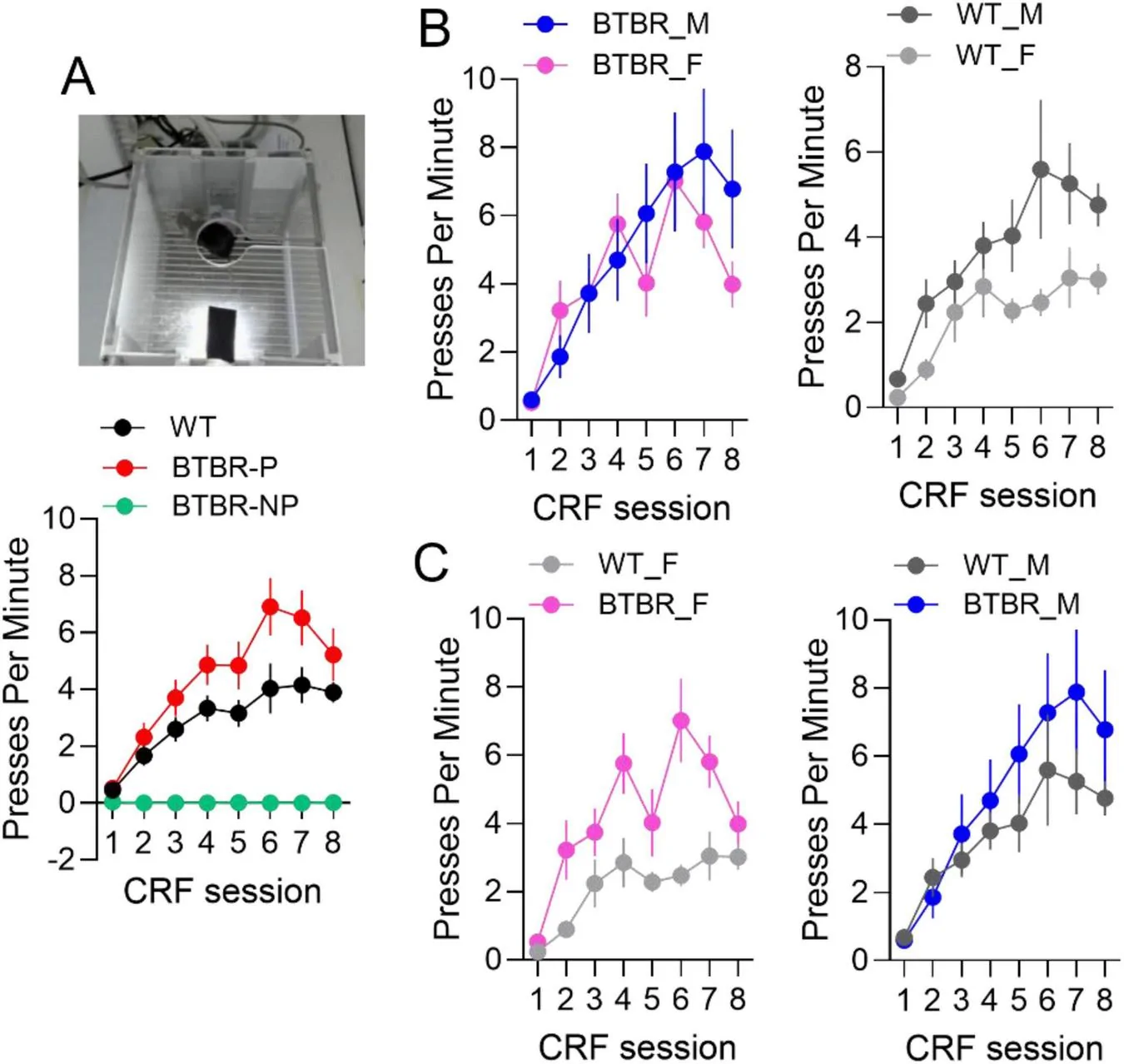
Initial acquisition of lever pressing under CRF in BTBR and WT mice. **(A)** Lever-press responses across eight CRF training sessions for mice that acquired the task. WT mice (*n* = 20) and most BTBR mice (BTBR-P, *n* = 16) successfully learned the task, while a subset of BTBR mice (BTBR-NP, *n* = 6) failed. Overall lever-press rates differed between BTBR and WT mice (*p* < 0.0001), but both genotypes showed comparable acquisition curves across sessions. Mice failed to acquire pressing were excluded from the comparisons **(B)** Sex-stratified analyses revealed similar acquisition patterns in BTBR males (*n* = 8) and females (*n* = 8, *p* = 0.29), whereas WT males (*n* = 10) exhibited higher lever-press rates than WT females (*n* = 10, *p* < 0.0001) during later sessions. **(C)** Sex-matched genotype comparisons showed that both female and male BTBR mice higher lever-press rates than their WT counterparts(females, *p* < 0.0001; males, *p* = 0.03). These findings indicate preserved instrumental acquisition and action-outcome learning in BTBR mice, despite sex- and genotype-dependent differences in response rates. Data are presented as mean ± SEM.

All mice were initially trained on a CRF schedule, in which each lever press was reinforced with a food pellet. All WT mice (*n* = 20) successfully acquired the lever-press response within eight training sessions. In the BTBR group, most mice (*n* = 16) acquired the task, whereas six mice failed to reach the acquisition criteria and were excluded from subsequent operant analyses. Among mice that acquired the task, a two-way mixed ANOVA revealed significant main effects of genotype (WT vs. BTBR, *F*_(1,288)_ = 20.4, *p* < 0.0001), and training session (*F*_(7,288)_ = 14.1, *p* < 0.0001), with no significant interaction between these factors was observed (*F*_(7,288)_ = 0.98, *p* = 0.45). These results indicate that, although BTBR mice exhibited overall higher lever-press rates than WT mice, both genotypes showed similar acquisition trajectories across training sessions, consistent with intact action–outcome learning in both strains (Figure 2A). Sex-stratified analyses revealed that BTBR males (*n* = 8) and females (*n* = 8) showed similar acquisition patterns, with a significant effect of session (*F*_(7,112)_ = 7.57, *p* < 0.0001), but no main effect of sex (*F*_(1,112)_ = 1.13, *p* = 0.29) or sex-session interaction (*F*_(7,112)_ = 0.91, *p* = 0.50) (Figure 2B). In contrast, WT males (*n* = 10) exhibited higher lever-press rates than WT females (*n* = 10) during later sessions (sex: *F*_(1,144)_ = 20.02, *p* < 0.0001; session: *F*_(7,144)_ = 7.16, *p* < 0.0001; interaction: *F*_(7,144)_ = 0.81, *p* = 0.58). Sex-matched genotype comparisons further revealed that female BTBR mice exhibited higher lever-press rates than female WT mice (Figure 2C; genotype: *F*_(1,128)_ = 42.15, *p* < 0.0001; session: *F*_(7, 128)_ = 9.62, *p* < 0.0001; interaction: *F*_(7,128)_ = 2.03, *p* = 0.057), with no significant interaction detected, suggesting similar acquisition trajectories across sessions. Similarly, BTBR males exhibited higher lever-press rates than WT males (genotype: *F*_(1,128)_ = 4.68, *p* = 0.03; session: *F*_(7,128)_ = 7.67, *p* < 0.0001; interaction: *F*_(7,128)_ = 0.53, *p* = 0.81). Together, these findings indicate that although BTBR mice exhibited consistently higher response rates during CRF training, both sexes acquired the lever-press response at comparable rates to WT controls.

### Habitual lever pressing

**FIGURE 3:**
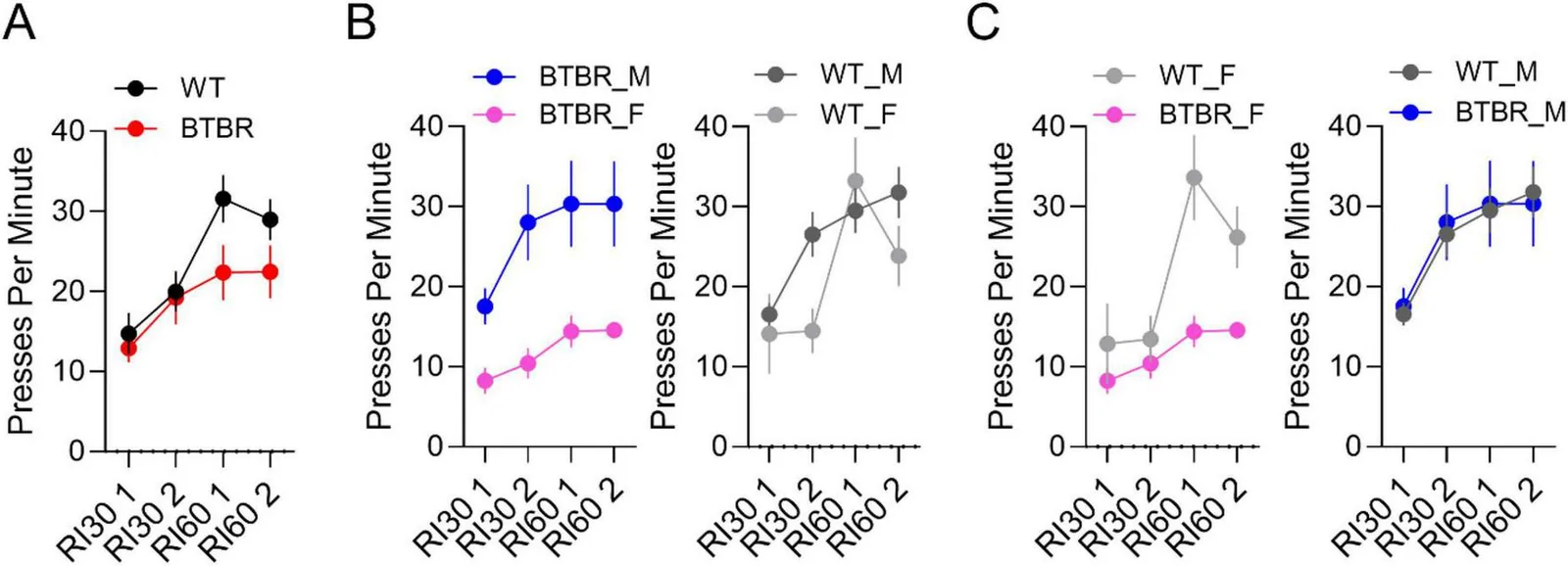
Lever pressing under RI schedules. **(A)** Lever-press responses across RI training sessions in BTBR (*n* = 16) and WT (*n* = 20) mice. Response rates increased across sessions in both genotypes, but BTBR mice exhibited lower overall lever-press rates compared with WT mice (*p* = 0.02). **(B)** Within-genotype sex analyses revealed that female BTBR mice (*n* = 8) exhibited lower lever-press rates than BTBR males (*n* = 8; *p* < 0.0001), whereas WT males (*n* = 10) and females (*n* = 10) showed comparable responses (*p* = 0.073). **(C)** Sex-matched genotype comparisons showed that female BTBR mice exhibited reduced lever pressing compared with WT females (*p* < 0.001), whereas male BTBR and WT mice did not differ (*p* = 0.86). These data indicate that while RI responding increased across training in both genotypes, female BTBR mice showed reduced engagement in habitual responding, suggesting sex-dependent alterations in the balance between goal-directed and habitual action control. Data are presented as mean ± SEM.

Following stabilization of initial acquisition, mice were transitioned to RI schedules to promote habitual responding. Lever-press performance increased across RI training sessions in both genotypes (session: *F*_(3,136)_ = 9.04, *p* < 0.0001), as indicated by a significant main effect of session However, BTBR mice exhibited overall lower response rates than WT mice (genotype: *F*_(1,136)_ = 5.14, *p* = 0.02), with no significant genotype-session interaction (*F*_(3,136)_ = 0.97, *p* = 0.41), indicating that both strains showed similar changes in responding across training despite differing overall response levels (Figure 3A). Within the BTBR mice, females exhibited significantly lower lever-press rates than males (sex: *F*_(1,56)_ = 35.6, *p* < 0.0001, session: *F*_(3,56)_ = 3.34, *p* = 0.03; interaction: *F*_(3,56)_ = 0.56, *p* = 0.64). In contrast, WT male and female mice showed no sex differences (*F*_(1,72)_ = 3.31, *p* = 0.073), with session effects reflecting general acquisition (*F*_(3,72)_ = 7.89, *p* = 0.001) (Figure 3B). Sex-matched comparisons indicated that female BTBR mice pressed less than WT females (genotype: *F*_(1,64)_ = 13.82, *p* < 0.001; session: *F*_(3,64)_ = 6.34, *p* < 0.001; interaction: *F*_(3,64)_ = 2.06, *p* = 0.11), whereas male BTBR and WT mice did not differ (genotype: F_(1,64)_ = 0.03, *p* = 0.86; session: *F*_(3,64)_ = 6.34, *p* < 0.001; interaction: *F*_(3,64)_ = 0.07, *p* = 0.98) (Figure 3C). Together, these findings demonstrate that habitual responding increased with RI training in both genotypes. However, the overall reduction in habitual lever pressing observed in BTBR mice was driven primarily by females, suggesting a sex-specific impairment in the development or expression of habitual responding.

### Outcome devaluation

**FIGURE 4:**
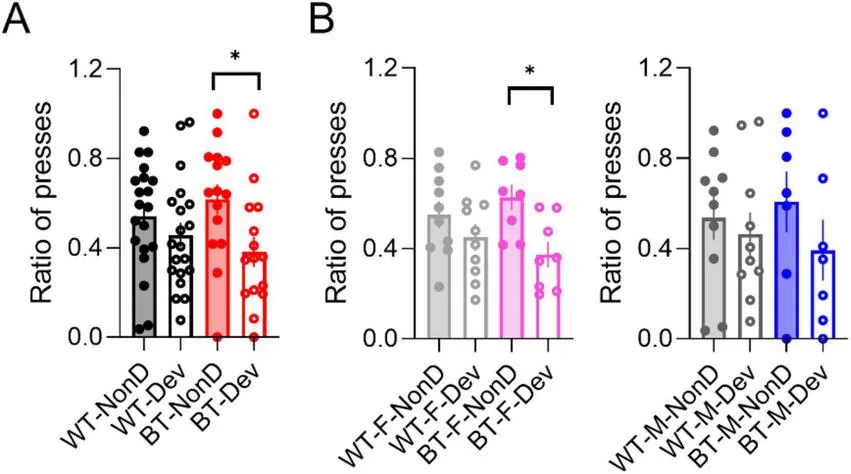
Outcome devaluation test. **(A)** Lever-press responses before and after outcome devaluation following RI training. WT mice (*n* = 20) showed no significant reduction in responding (*p* = 0.17), whereas BTBR mice (*n* = 15) significantly reduced lever pressing (*p* = 0.032). **(B)** Sex-stratified analyses showed significant devaluation sensitivity in female BTBR mice (*n* = 8, *p* = 0.039), but not male BTBR mice (*n* = 7, *p* = 0.25). WT males and females showed no significant changes after devaluation (10 males: *p* = 0.37; 10 females: *p* = 0.25). These findings indicate enhanced sensitivity to action-outcome contingencies in BTBR mice, particularly females. Data are presented as mean ± SEM. * *p* < 0.05.

To assess sensitivity to action-outcome contingencies following extended RI training, mice were subjected to outcome devaluation test. WT mice showed no significant reduction in lever pressing following devaluation (Wilcoxon signed-rank test, *p* = 0.17), consistent with habitual control. In contrast, BTBR mice exhibited a significant reduction (*p* = 0.032), indicating that their behavior remained sensitive to outcome value and therefore retained goal-directed control (Figure 4A). Sex-stratified analyses revealed that both male and female BTBR mice exhibited greater sensitivity to outcome devaluation than their WT counterparts (Figure 4B). This effect reached statistical significance in BTBR females (*p* = 0.039) but not in BTBR males (*p* = 0.25). In contrast, WT mice of both sexes showed minimal devolution sensitivity (males: *p* = 0.37; females: *p* = 0.25). Together, these findings demonstrate that BTBR mice, particularly females, exhibit impaired habit formation, as evidenced by both reduced habitual responding during RI training and persistent sensitivity to outcome devaluation.

### Omission test

**FIGURE 5:**
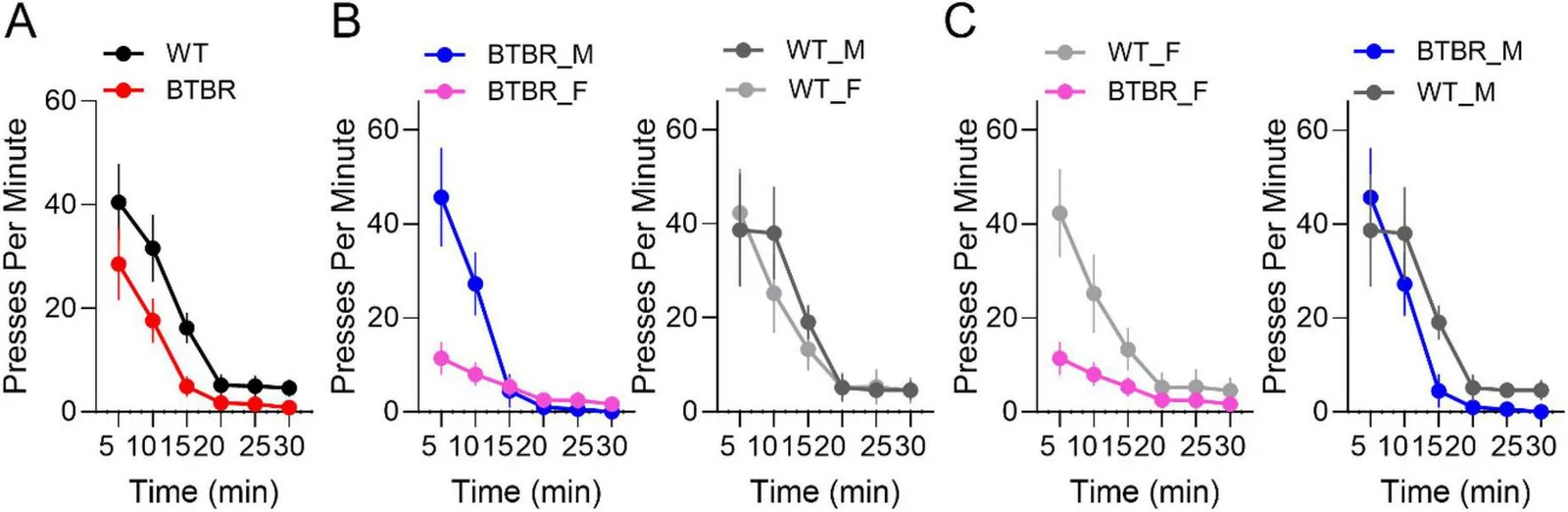
Omission test for action-outcome sensitivity. **(A)** Across all mice (BTBR, *n* = 16; WT, *n* = 20), lever pressing decreased over time during the omission test, with significant main effects of time (*p* < 0.0001) and genotype (*p* = 0.001), indicating that BTBR mice suppressed responding more readily than WT controls. **(B)** Within BTBR mice, females (*n* = 8) showed a faster reduction in lever pressing during the omission test, with significant suppression occurring within the first 10 min compared with BTBR males (*n* = 8, *ps* < 0.01). No sex differences were observed in WT mice (males, *n* = 10; females, *n* = 10, *p* = 0.52). **(C)** Sex-matched genotype comparisons demonstrated that female BTBR mice reduced responding faster than WT females (*p* = 0.03), whereas male BTBR and WT mice did not differ (*p* = 0.15). These findings indicate enhanced sensitivity to action-outcome contingencies in female BTBR mice. Data are presented as mean ± SEM.

To determine whether goal-directed control extended to contingency reversal, mice were tested under an omission schedule where lever pressing prevented reward delivery. A two-way mixed ANOVA revealed significant main effects of time (F_(5,204)_ = 21.07, *p* < 0.0001) and genotype (F_(1,204)_ = 11.2, *p* = 0.001), with no time-genotype interaction (F_(5,204)_ = 0.72, *p* = 0.61), indicating that BTBR mice suppressed responding more readily than WT controls (Figure 5A). Within the BTBR group, a significant time-sex interactions were observed (F_(5,84)_ = 7.12, *p* < 0.0001). *Post hoc* analyses revealed that female BTBR mice rapidly reduced responding during the first 10 min of the omission session (*p* < 0.0001 and *p* < 0.01), demonstrating greater sensitivity to reversed action-outcome contingencies than BTBR males (Figure 5B). In contrast, WT males and females exhibited similar response patterns, with no significant main effect of sex (F_(1,108)_ = 0.42, *p* = 0.52). Sex-matched comparisons further demonstrated that female BTBR mice reduced lever pressing more rapidly than WT females, as evidenced by a significant time-sex interactions (F_(5,96)_ = 2.66, *p* = 0.03), *Post hoc* analyses showed that BTBR females responded significantly less than WT females within the first 5 min of the omission session (*p* = 0.0001). In contrast, no genotype differences were observed between BTBR and WT males (F_(1,96)_ = 2.06, *p* = 0.15) (Figure 5C). Together, these findings demonstrate that BTBR mice, particularly females, adapt more rapidly to reversed action–outcome contingencies, indicating persistent goal-directed control despite extensive habit training. These results are consistent with the outcome devaluation data and further support a selective impairment in the transition from goal-directed to habitual action control in female BTBR mice.

### Rotarod performance

**FIGURE 6:**
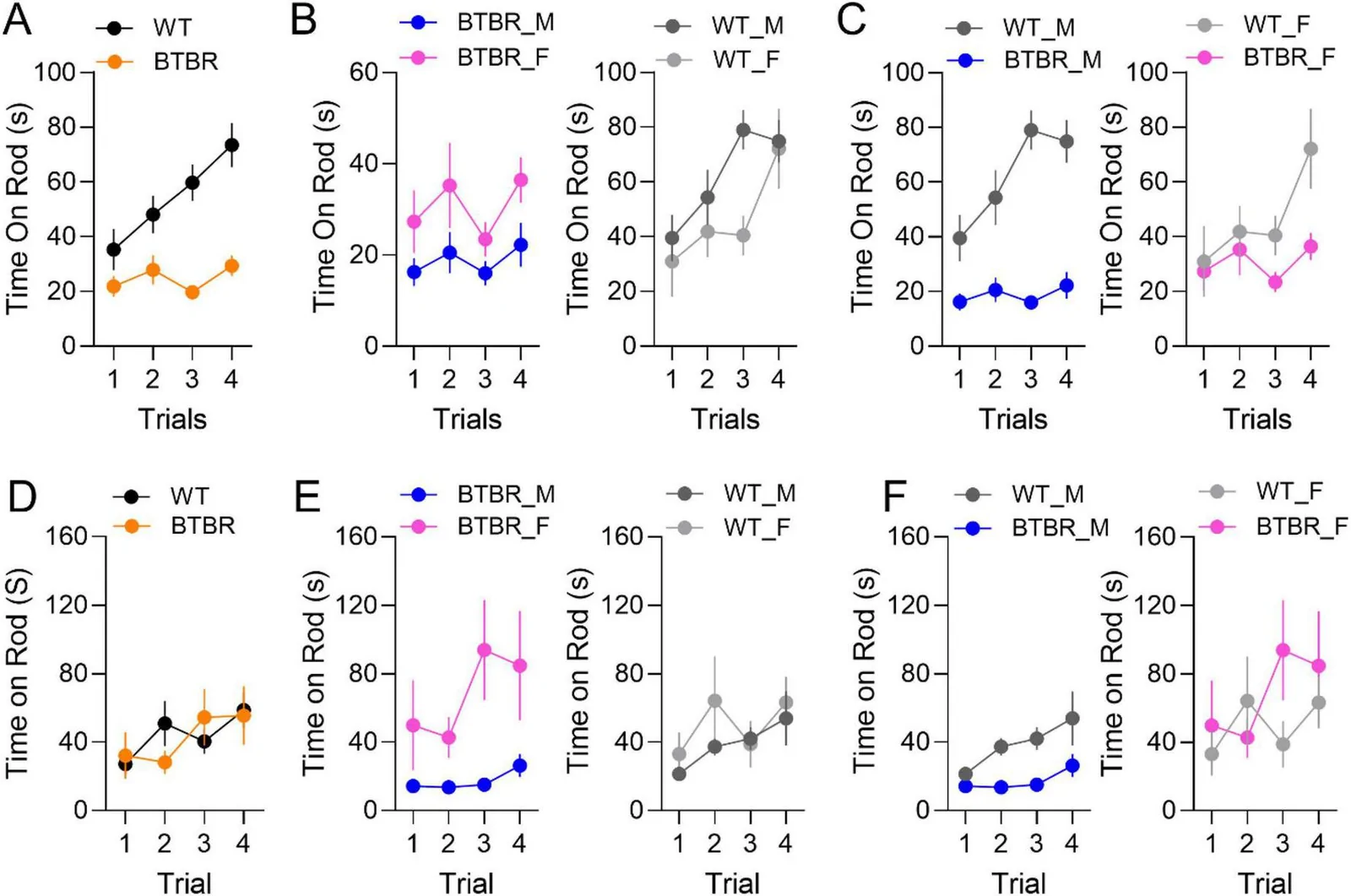
Rotarod performance in BTBR and WT mice. **(A)** During accelerating rotarod testing on Day 1, BTBR mice (*n* = 22) exhibited impaired motor learning compared with WT controls (*n* = 20). WT mice improved across trials, whereas BTBR mice failed to show significant improvement, indicating reduced adaptation to increasing motor demands. BTBR mice that acquired and failed to acquire the operant task showed comparable rotarod performance (all *ps* > 0.05) and were combined for subsequent analyses. **(B)** Within-genotype sex analyses revealed lower performance in BTBR males (*n* = 11) compared with BTBR females (*n* = 11; *p* = 0.003), whereas WT males (*n* = 10) showed higher overall performance than WT females (*n* = 10; *p* = 0.03). **(C)** Sex-matched comparisons demonstrated impaired motor learning in BTBR males compared with WT males (*p* < 0.0001), whereas female BTBR and WT mice showed comparable learning trajectories. **(D)** During constant speed rotarod testing on Day 2, performance stabilized across trials, with no overall genotype difference (*p* = 0.85), indicating preserved baseline motor coordination. **(E)** Within-genotype sex analyses showed reduced performance in BTBR males compared with BTBR females (*p* < 0.001), whereas WT males and females did not differ (*p* = 0.26). **(F)** Sex-matched comparisons revealed reduced performance in BTBR males compared with WT males (*p* < 0.001), while BTBR and WT females showed comparable performance (*p* = 0.27). Together, these findings indicate a male-predominant motor phenotype in BTBR mice characterized by impaired motor learning under increasing task demands, with relatively preserved baseline coordination. Data are presented as mean ± SEM.

Motor coordination and learning were assessed using an accelerating rotarod across two consecutive days. On Day 1, mice completed four accelerating trials designed to evaluate motor learning under progressively increasing task demands. Overall, BTBR mice exhibited impaired motor learning compared with WT mice (Figure 6A). A two-way ANOVA revealed significant main effects of genotype (F_(1,160)_ = 52.7, *p* < 0.0001) and trial (F_(3,160)_ = 5.37, *p* = 0.002), as well as a significant trial-genotype interaction (F_(3,160)_ = 3.40, *p* = 0.019). *Post hoc* analyses indicated that WT mice (*n* = 20) significantly improved their performance across trials (*ps* < 0.05), whereas BTBR mice (*n* = 22) showed no improvement (all *ps* > 0.05), indicating impaired motor learning rather than simply reduced motor performance. Because BTBR mice that acquired the operant task and those that failed to acquire it exhibited comparable rotarod performance (all *ps* > 0.05), these groups were combined for subsequent analyses. Sex-stratified analyses indicated that the motor learning deficit was more pronounced in males (Figure 6B). Within the BTBR group, males (*n* = 11) performed significantly worse than females (*n* = 11), reflected by a significant main effect of sex (F_(1,80)_ = 19.9, *p* = 0.003). However, neither BTBR males nor females exhibited significant within-group improvements across the four training trials (all *ps* > 0.05). In contrast, both WT males (*n* = 10) and WT female (*n* = 10) significantly improved across trials, with WT males achieving higher overall performance than WT female (sex, F_(1,72)_ = 4.79, *p* = 0.03; trial, F_(3,72)_ = 5.28, *p* = 0.02; sex-trial, F_(3,72)_ = 1.24, *p* = 0.30). Sex-matched genotype comparisons further demonstrated that the motor learning deficit was predominantly male-driven (Figure 6C). In males, significant main effects of genotype (F_(1,76)_ = 91.4, *p* < 0.0001) and trial (F_(3,76)_ = 4.63, *p* = 0.005), together with a significant genotype-trial interaction (F_(3,76)_ = 3.96, *p* = 0.01) indicated impaired motor learning in BTBR mice. *Post hoc* comparisons confirmed that WT males improved across trials (*ps* < 0.05), whereas BTBR males did not (all *ps* > 0.05). In females, significant main effect of genotype (F_(1,76)_ = 5.99, *p* = 0.017) and trial (F_(3,76)_ = 3.05, *p* = 0.03) were observed, but the absence of genotype-trial interaction (F_(3,76)_ = 1.26, *p* = 0.29) indicates that the pattern of performance across trials was similar between BTBR and WT females.

To determine whether these deficits reflected impaired motor learning or baseline motor coordination, mice were tested on Day 2 at a constant rotation speed rotarod. Under steady-speed conditions, performance stabilized across trials, with no significant effect of trial (*F*_(3,160)_ = 1.76, *p* = 0.16), genotype (*F*_(1,160)_ = 0.04, *p* = 0.85), or interaction (*F*_(3,160)_ = 0.79, *p* = 0.50), indicating that the strain differences observed on Day 1 were specific to the accelerating task (Figure 6D). As on Day 1, BTBR mice that acquired the operant task and those that failed to acquire it did not differ in rotarod performance (all *ps* > 0.05) and were therefore combined for subsequent analyses. Within the BTBR group, females again outperformed males, reflected by a significant main effect of sex (*F*_(1,80)_ = 14.6, *p* < 0.001), with no significant effects of trial (*F*_(3,80)_ = 1.19, *p* = 0.32) or interaction effects (*F*_(3,80)_ = 0.74, *p* = 0.53). In contrast, WT males and females performed similarly, with no significant main effect of sex (*F*_(1,72)_ = 1.29, *p* = 0.26), trial (*F*_(3,72)_ = 1.88, *p* = 0.14) or interaction (*F*_(3,72)_ = 0.39, *p* = 0.76) (Figure 6E). Sex-matched genotype comparisons further showed that BTBR males exhibited lower overall performance than WT males (genotype, *F*_(1,76)_ = 18.4, *p* < 0.001), with a significant effect of trial (*F*_(3,76)_ = 3.44, *p* = 0.02), but no interaction (*F*_(3,76)_ = 0.95, *p* = 0.42). In contrast, BTBR and WT females performed comparably, with no significant effects of genotype (*F*_(1,76)_ = 1.25, *p* = 0.27), trial (*F*_(3,76)_ = 0.80, *p* = 0.50) or interaction (*F*_(3,76)_ = 0.95, *p* = 0.42) (Figure 6F). Together, these findings demonstrate that BTBR mice exhibit impaired performance on the accelerating rotarod, with deficits that are more pronounced in males. The absence of genotype differences during constant-speed testing indicates that the primary impairment is associated with adaptation to increasing motor demands rather than a generalized deficit in motor coordination, although BTBR males exhibit modest reductions in overall motor performance.

### Social interaction

**FIGURE 7:**
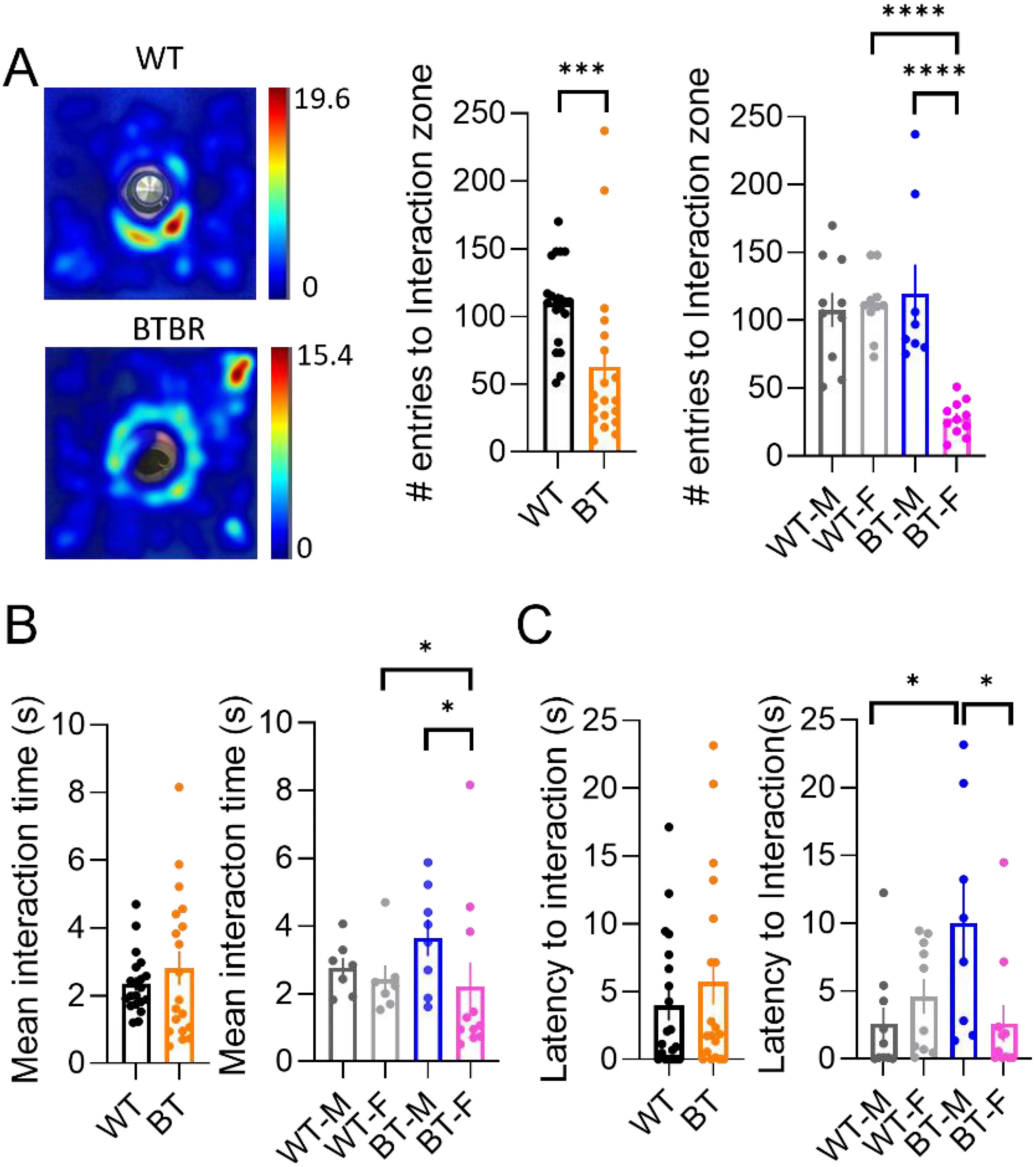
Sex-specific alterations in social approach behavior in BTBR mice. **(A)** Left, representative heatmaps showing movement trajectories during the social interaction test. Right, BTBR mice (*n* = 19) exhibited fewer entries into the interaction zone compared with WT controls (*n* = 20; *p* < 0.001). Sex-stratified analyses revealed that this reduction was primarily driven by females: BTBR females (*n* = 11) showed fewer interaction zone entries than BTBR males (*n* = 9) (*p* < 0.0001) and WT females (*n* = 10, *p* < 0.0001), whereas WT mice showed no sex difference (*p* = 0.32). **(B)** Total time spent in the interaction zone did not differ between BTBR and WT mice (*p* = 0.40). However, within-strain comparisons showed that BTBR females spent less time interacting than BTBR males (*p* = 0.016) and WT females (*p* = 0.042), while WT mice showed no sex difference (*p* = 0.32). **(C)** Latency to first investigation was not different between genotypes overall (*p* = 0.24); however, BTBR males showed longer investigation latencies compared with BTBR females (*p* = 0.016) and WT males (*p* = 0.015). Together, these findings indicate that social alterations in BTBR mice are sex- and dimension-specific, with reduced social engagement more prominent in females and delayed initiation of social investigation observed in males. Data are presented as mean ± SEM. *p < 0.05; ***p < 0.001; ****p < 0.0001.

Social interaction was assessed by quantifying the number of entries into the interaction zone, total time spent in the interaction zone, and latency to first investigation. BTBR mice (*n* = 19) exhibited reduced social approach behavior compared with WT controls (*n* = 20), as reflected by significantly fewer entries into interaction zone (Figure 7A, Mann-Whitney test, *p* < 0.001). Within the BTBR strain, females (*n* = 11) showed significantly fewer interaction zone entries than males (*n* = 8, *p* < 0.0001), whereas no sex differences were observed in WT female (*n* = 10) and male (*n* = 10) mice (*p* = 0.32). Sex-matched genotype comparisons revealed no significant difference between WT and BTBR males (*p* = 0.50), while BTBR females exhibited significantly fewer interaction zone entries than WT females (*p* < 0.0001). These findings indicate that the reduction in social approach behavior was driven primarily by female BTBR mice. Analysis of total interaction time revealed a similar pattern (Figure 7B). Overall, BTBR and WT mice did not differ in the total time spent in the interaction zone (*p* = 0.40). However, within the BTBR group, females spent significantly less time interacting than males (*p* = 0.016). Likewise, BTBR females spent less time in the interaction zone than WT females (*p* = 0.042), whereas no significant difference was observed between BTBR and WT males (*p* = 0.17). No sex difference was detected in WT mice (*p* = 0.32). Together, these findings further support a female-specific reduction in social engagement. In contrast, latency to first investigation exhibited a distinct pattern (Figure 7C). No overall genotype difference was detected (*p* = 0.24). However, BTBR males displayed significantly longer latencies to initiate social investigation than BTBR females (*p* = 0.016) and WT males (*p* = 0.015). Latency did not differ between BTBR and WT females (*p* = 0.15) or between WT males and WT females (*p* = 0.15). Collectively, these findings indicate that social impairments in BTBR mice are sex- and measure-dependent. Female BTBR mice exhibit reduced social engagement, reflected by fewer interaction zone entries and less time spent interacting, whereas male BTBR mice display delayed initiation of social investigation despite otherwise normal levels of social engagement.

### Anxiety-like behavior

**FIGURE 8:**
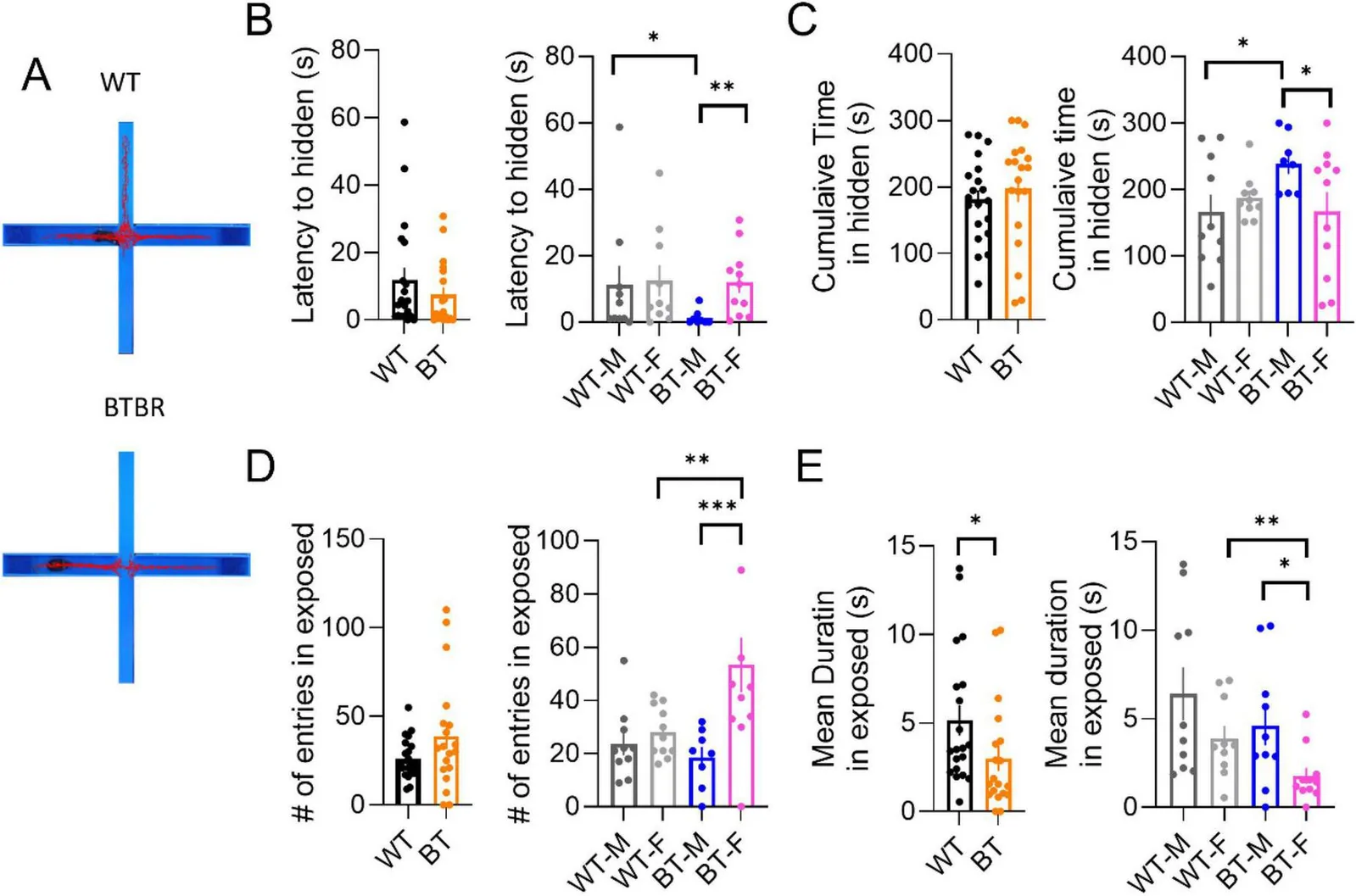
Sex-dependent anxiety-like behavior in EPM. **(A)** Representative movement trajectories of WT and BTBR mice during EPM testing. **(B)** Latency to enter the closed arms did not differ between BTBR (*n* = 19) and WT (*n* = 20) mice (*p* = 0.44). However, BTBR males (*n* = 8) entered the closed arms faster than BTBR females (*n* = 11; *p* = 0.002) and WT males (*n* = 10; *p* = 0.016), whereas WT mice showed no sex difference (*p* = 0.128). **(C)** Cumulative time spent in the closed arms did not differ between genotypes (*p* = 0.13). BTBR males spent more time in the closed arms than BTBR females (*p* = 0.033) and WT males (*p* = 0.020). **(D)** Open-arm entries did not differ between genotypes (*p* = 0.23). BTBR females exhibited more open-arm entries than BTBR males (*p* < 0.001) and WT females (*p* = 0.008), whereas WT mice showed no sex difference (*p* = 0.28). **(E)** Mean duration in the open arms did not differ between genotypes (*p* = 0.30). BTBR females spent less time in the open arms than BTBR males (*p* = 0.022) and WT females (*p* < 0.01), whereas BTBR males were comparable to WT males (*p* = 0.30). Together, these findings indicate that anxiety-related and exploratory behaviors in BTBR mice are sex dependent, with males displaying a more avoidant profile and females showing increased but less sustained open-arm exploration. Data are presented as mean ± SEM. *p < 0.05; **p < 0.01; ***p < 0.001.

Anxiety-like behavior was assessed using EPM by measuring latency to enter the closed arms, cumulative time spent in the closed arms, the number of open-arm entries, and the mean duration in the open arms (Figure 8A). Across genotypes, BTBR (*n* = 19) and WT (*n* = 20) mice did not differ in latency to enter the closed arms (Figure 8B, *p* = 0.44). However, within the BTBR strain, males (*n* = 8) entered the closed arms significantly sooner than BTBR females (*n* = 11, *p* = 0.002) and WT males (*n* = 10, *p* = 0.016), whereas WT males and females (*n* = 10) did not differ (*p* = 0.295). Similarly, total time spent in the closed arms did not differ between BTBR and WT mice overall (Figure 8C, *p* = 0.13). However, BTBR males spent significantly more time in the closed arms than both BTBR females (*p* = 0.033) and WT males (*p* = 0.02), while no sex difference was observed in WT mice (*p* = 0.23). The number of open-arm entries also did not differ genotypes (Figure 8D, *p* = 0.152). However, within the BTBR stain, females made significantly more open-arm entries than BTBR males (*p* < 0.001) and WT females (*p* = 0.008). No sex difference was observed in WT mice (*p* = 0.278). In contrast, BTBR mice spent significantly less time in the open arms than WT controls (Figure 8E, *p* = 0.011). This effect was driven primarily by females, as BTBR females spent less time in the open arms than both BTBR males (*p* = 0.022) and WT females (*p* < 0.01), whereas BTBR and WT males did not differ (*p* = 0.30). Collectively, these findings reveal sex-specific patterns of anxiety-related behavior in BTBR mice. BTBR males exhibited greater avoidance of the open arms, reflected by more rapid entry into and prolonged occupancy of the closed arms. In contrast, BTBR females entered the open arms more frequently but remained there for shorter periods, suggesting altered exploratory behavior or risk-assessment strategies rather than a uniform change in anxiety-like behavior.

## Discussion

In this study, we conducted a systematic, multi-domain behavioral assessment of male and female BTBR mice to determine how sex influences the expression of autism-relevant phenotypes. Across operant learning, action control, motor function, social behavior, and anxiety-related exploration, BTBR mice exhibited distinct, domain-specific, and sex-dependent behavioral alterations rather than a uniform phenotype (Table 1). Instrumental learning was preserved in both sexes; however, female BTBR mice exhibited impaired habit formation, persistent goal-directed control, reduced social engagement, and altered exploratory behavior, whereas male BTBR mice showed more pronounced deficits in motor learning, delayed initiation of social investigation, and greater avoidance-related behavior. Together, these findings demonstrate that sex influences not only the severity but also the behavioral manifestation of ASD-related phenotypes, underscoring the importance of incorporating sex as a biological variable in preclinical ASD research.

**TABLE 1:** Summary of sex-dependent behavioral phenotypes in BTBR mice.

Domain	Male BTBR	Female BTBR
Instrumental learning	Normal	Normal
Habit formation	Mild impairment	Pronounced impairment
Goal-directed control	Mild increase	Persistent
Motor learning	Pronounced impairment	Mild impairment
Baseline motor coordination	Mild deficit	Normal
Social interaction	Delayed investigation	Reduced engagement
Anxiety/EPM	Avoidant behavior	Altered exploration

### Altered action control and habit formation

Operant testing revealed altered action control in BTBR mice, with a prominent sex-dependent effect in females. BTBR mice displayed intact instrumental acquisition under continuous reinforcement, indicating preserved action-outcome learning (Balleine and Dickinson, 1998). Although response rates differed by genotype and sex, the absence of genotype-session interactions suggest comparable learning across strains. Following extended training under RI schedules that typically promote habit formation (Dickinson et al., 1983; Yu et al., 2009), female BTBR mice exhibited reduced lever pressing compared with WT females, suggesting altered expression of habitual responding. Consistent with this interpretation, BTBR mice remained greater sensitive to outcome devaluation, whereas WT mice showed the expected shift toward habitual control after extended training (Yu et al., 2009). Female BTBR mice also demonstrated more rapid suppression during the omission test, indicating enhanced sensitivity to action-outcome contingencies. Together, these findings suggest that BTBR mice, particularly females, show a reduced transition from goal-directed to habitual control, resulting in persistent goal-directed regulation of behavior. Altered balance between goal-directed and habitual systems has been implicated in neurodevelopmental disorders, including ASD (Robbins et al., 2012; Gillan et al., 2016). The sex-dependent differences observed here may reflect altered regulation of corticostriatal circuits, including interactions between dorsomedial striatum (DMS) circuits supporting goal-directed behavior and dorsolateral striatum (DLS) circuits mediating habitual responding (Yin et al., 2004; Yin and Knowlton, 2006; Yu et al., 2009; Gremel and Costa, 2013). Altered prefrontal-striatal connectivity or striatal plasticity could underlie the reduced habit formation in BTBR mice, and sex differences may involve modulatory effects of gonadal hormones on these circuits (Becker et al., 2016). Overall, these findings link persistent goal-directed behavior in BTBR mice to potential circuit-level mechanisms contributing to ASD-relevant behavioral rigidity.

### Male-specific motor learning vulnerability

Rotarod testing revealed that BTBR mice exhibit impaired motor learning under accelerating conditions, with deficits primarily driven by males, whereas performance under constant-speed conditions indicated largely preserved baseline motor coordination. These findings suggest that male BTBR mice exhibit reduced adaptation to increasing motor demands rather than a generalized impairment in motor execution. The sex-specific nature of this phenotype is consistent with reports of male-biased motor abnormalities in ASD mouse models (Wang et al., 2016; Cording and Bateup, 2023), highlighting the importance of including both sexes in preclinical studies. Female BTBR mice showed performance comparable to WT females, suggesting relative resilience in motor learning under dynamic task demands. Motor learning during rotarod performance depends on coordinated activity across cerebellar, basal ganglia, and corticostriatal circuits (Yin et al., 2009; D’Angelo and Casali, 2012). Thus, the male-specific deficits observed in BTBR mice may reflect altered cerebellar function, impaired corticostriatal plasticity, or disrupted interactions between motor control networks, consistent with evidence of cerebellar abnormalities and striatal dysfunction in ASD models (Wang et al., 2014; D’Mello and Stoodley, 2015). The relative preservation of constant-speed performance further suggests that dynamic motor adaptation, rather than basic motor coordination, is selectively affected. Sex-dependent regulation of cerebellar and corticostriatal circuits, potentially influenced by gonadal hormones, may contribute to this male vulnerability (Becker et al., 2016). Overall, these findings identify a male-predominant deficit in motor learning under increasing task demands and provide a behavioral framework for investigating sex-specific mechanisms underlying motor dysfunction in ASD.

### Sex-divergent components of social dysfunction in BTBR mice

Social interaction testing revealed that BTBR mice exhibit sex- and dimension-specific alterations in social behavior. Across genotypes, BTBR mice showed reduced social approach, reflected by fewer entries into the interaction zone, although total interaction time did not differ between genotypes. Sex-stratified analyses further demonstrated that these effects were primarily driven by females: BTBR females exhibited reduced interaction zone entries and decreased time spent in the interaction zone, whereas BTBR males showed delayed initiation of social investigation without reductions in overall interaction measures. These findings are consistent with previous studies demonstrating impaired social approach and reduced social exploration in BTBR mice (McFarlane et al., 2008; Moy et al., 2008; Silverman et al., 2010). The distinct behavioral profiles observed between sexes suggest that social alterations in BTBR mice are not uniform but depend on the specific component of social behavior being assessed. This domain-specific pattern parallels clinical observations that ASD-related social difficulties can vary across individuals and may differ between males and females (Werling and Geschwind, 2013; Lai et al., 2015). The sex-dependent differences identified here may reflect differential regulation of neural circuits involved in social motivation, approach, and social initiation. Social behavior relies on coordinated interactions among the prefrontal cortex, amygdala, and nucleus accumbens, and disruptions within these networks have been implicated in ASD-related social phenotypes (Panksepp and Lahvis, 2007; Sato et al., 2023). Sex-dependent modulation of these circuits, potentially through gonadal hormone signaling or differences in synaptic plasticity, may contribute to the female-predominant reduction in social engagement and male-specific delay in social investigation (Becker et al., 2016; Bove et al., 2024). Overall, these results highlight that social deficits in BTBR mice are both sex- and dimension-specific, emphasizing the importance of analyzing multiple behavioral parameters and including both sexes in preclinical ASD research.

### Sex-specific anxiety-like behavior

In the EPM, BTBR mice did not exhibit robust genotype-dependent differences across anxiety-related measures. Open-arm entries, time spent in the open arms, and latency to enter the closed arms were largely comparable between BTBR and WT mice, suggesting that the BTBR strain does not display a strong global increase in anxiety-like behavior under these conditions. However, sex-dependent behavioral patterns emerged within the BTBR group. BTBR males entered the closed arms more rapidly and spent more time in the closed arms, consistent with a more avoidant exploratory profile. In contrast, BTBR females exhibited increased open-arm entries but reduced time spent in the open arms, suggesting increased environmental sampling with reduced sustained exploration of exposed areas. These findings indicate a dissociation between exploratory activity and avoidance-related behavior, with BTBR males and females exhibiting distinct behavioral strategies rather than a uniform anxiety phenotype. The frequent but brief open-arm visits observed in females may reflect altered risk assessment or exploratory sampling, whereas the prolonged closed-arm preference in males suggests increased avoidance of exposed environments. Similar sex-dependent differences in exploration and avoidance strategies have been reported in anxiety-related behavioral paradigms, with males and females showing distinct responses to environmental challenge (Johnston and File, 1991; Knight et al., 2021). Overall, these results indicate that anxiety-related behavior in BTBR mice is subtle, context dependent, and strongly influenced by sex. These findings emphasize the importance of analyzing multiple behavioral parameters rather than relying on single EPM measures to define anxiety phenotypes in ASD-relevant models. A more refined assessment of behavioral strategy and sex-specific patterns may provide greater insight into the neural mechanisms underlying ASD-associated alterations in emotional regulation and exploration.

### Integrated interpretation across domains

BTBR mice exhibit a domain- and sex-dependent profile of ASD-relevant behavioral alterations rather than a uniform phenotype. Previous studies have demonstrated that BTBR mice exhibit abnormalities in corticostriatal and cortico-limbic circuitry, impaired dopaminergic neurotransmission, cerebellar dysfunction, and altered synaptic signaling, which are neural systems implicated in action selection, motor learning, social behavior, and behavioral flexibility (Squillace et al., 2014; Meyza and Blanchard, 2017; Kiffmeyer et al., 2022). These established neurobiological features provide a potential framework for interpreting the behavioral phenotypes observed in the present study, although the underlying mechanisms were not directly examined here. Across behavioral domains, male and female mice displayed distinct patterns of vulnerability. Male BTBR mice showed more pronounced deficits in motor learning, delayed initiation of social investigation, and increased avoidance-related behavior, suggesting altered adaptation and behavioral responses during challenging or socially relevant contexts. In contrast, female BTBR mice exhibited impaired transition toward habitual responding, persistent goal-directed control, reduced social engagement, and altered exploratory patterns in the elevated plus maze, reflecting sex-specific differences in action selection and environmental exploration. Importantly, anxiety-related measures showed limited genotype-wide effects but revealed distinct sex-dependent exploratory strategies, emphasizing that behavioral alterations in BTBR mice cannot be fully captured by aggregate measures alone. Instead, ASD-relevant phenotypes in this model appear to reflect shifts in behavioral strategy, including action selection, exploration, social engagement, and adaptation to changing demands. These sex-specific behavioral profiles may arise from differential regulation of corticostriatal, cortico-limbic, and motor control circuits, potentially influenced by sex-dependent developmental mechanisms and hormonal modulation. In particular, sex differences in dopaminergic signaling, synaptic plasticity, and developmental regulation of neural circuits may contribute to the divergent behavioral strategies observed between male and female BTBR mice. However, these potential mechanisms remain to be directly tested and will require future studies integrating behavioral analysis with circuit-specific electrophysiology, neurochemical measurements, and molecular approaches. Together, these findings highlight that BTBR mice do not exhibit a single ASD-like behavioral phenotype; rather, they display distinct sex- and domain-specific alterations that may better model the heterogeneity of ASD. Incorporating sex as a biological variable is therefore essential for improving the interpretation, mechanistic understanding, and translational relevance of preclinical ASD studies.

### Limitations and future directions

Several limitations should be considered when interpreting the present findings. First, this study was designed to systematically characterize behavioral phenotypes and therefore did not directly investigate the neural mechanisms underlying the observed sex-dependent differences. Multiple biological factors may contribute to these divergent behavioral profiles, including sex-dependent regulation of corticostriatal and cortico-limbic circuits, alterations in dopamine signaling, differences in synaptic plasticity, and the organizational and activational effects of gonadal hormones during development. Determining the relative contributions of these mechanisms will require future studies that integrate behavioral analyses with electrophysiological recordings, neurotransmitter measurements, molecular profiling, and circuit-specific manipulations. Second, although the BTBR mouse is a well-established preclinical model of ASD, no single animal model fully captures the biological and clinical heterogeneity of the disorder. The complete agenesis of the corpus callosum in BTBR is an important model-specific feature that may influence some of the behavioral phenotypes observed in this strain. Therefore, the present findings should be interpreted within the context of this model, and this neuroanatomical feature should be considered when evaluating the generalizability and translational relevance of the findings. Future studies should extend these findings across complementary genetic and environmental models of ASD and combine behavioral phenotyping with circuit-level and molecular investigations. Such approaches will help determine which sex-dependent behavioral phenotypes are conserved across models and identify their underlying neural mechanisms, ultimately improving our understanding of sex-dependent ASD pathophysiology and strengthening the translational relevance of preclinical studies.

## Data Availability

The raw data supporting the conclusions of this article will be made available by the authors, without undue reservation.
